# Correlation of Solvent Interaction Analysis Signatures with Thermodynamic Properties and In Silico Calculations of the Structural Effects of Point Mutations in Two Proteins

**DOI:** 10.3390/ijms25179652

**Published:** 2024-09-06

**Authors:** Amber R. Titus, Pedro P. Madeira, Vladimir N. Uversky, Boris Y. Zaslavsky

**Affiliations:** 1 Cleveland Diagnostics, 3615 Superior Ave., Cleveland, OH 44114, USA; amber.titus@clevelanddx.com (A.R.T.); p.madeira@ua.pt (P.P.M.); 2 CICECO—Aveiro Institute of Materials, Department of Chemistry, University of Aveiro, 3810-193 Aveiro, Portugal; 3 Department of Molecular Medicine and Byrd Alzheimer’s Research Institute, Morsani College of Medicine, University of South Florida, Tampa, FL 33612, USA

**Keywords:** aqueous two-phase system, protein partitioning, solvent interaction analysis, staphylococcal nuclease A, bacteriophage T4 lysozyme, conformational stability, structural signature

## Abstract

The partition behavior of single and double-point mutants of bacteriophage T4 lysozyme (T4 lysozyme) and staphylococcal nuclease A was examined in different aqueous two-phase systems (ATPSs) and studied by Solvent Interaction Analysis (SIA). Additionally, the solvent accessible surface area (SASA) of modeled mutants of both proteins was calculated. The in silico calculations and the in vitro analyses of the staphylococcal nuclease and T4 lysozyme mutants correlate, indicating that the partition analysis in ATPSs provides a valid descriptor (SIA signature) covering various protein features, such as structure, structural dynamics, and conformational stability.

## 1. Introduction

When two nonionic polymers (e.g., dextran, Ficoll, polyethylene glycol, etc.) or a single polymer and phase-forming salt/organic compound are mixed in water above certain concentration thresholds, they form biphasic systems. These aqueous two-phase systems (ATPSs) contain two partially immiscible coexisting aqueous phases, each consisting of ca. 80 mol% water and preferentially enriched in one of the phase-forming components (polymer(s)/salt/organic compound). For polymer–polymer and polymer–salt ATPSs, the overall polymer composition of the mixture defines the concentrations of each polymer in both phases, whereas the salt compositions of the phases are governed by the type and concentration of the salt additive, which is also related to the overall polymer composition [1]. Dielectric, partition, potentiometric, and solvatochromic measurements have demonstrated [1,2,3] that the solvent features of water differ in polymer solutions and depend on the type, concentration, and molecular weight of the polymer. It is also well established that electrostatic, hydrophobic, and other solvent properties of aqueous media [2,3,4] differ between the two co-existing phases in a given ATPS. The differences between the physicochemical properties of two aqueous phases in an ATPS are small, relative to those found in organic solvent–water systems. However, these small differences have been shown to govern the partition behavior of various solutes, which can range from simple organic compounds to proteins, nucleic acids, and even cells [3]. The phase-forming polymers used in ATPSs are not engaged in direct interactions with a solute, but the distribution of a given solute between the phases is driven by the solvent properties of the aqueous phases. Since the solute–solvent interactions govern the partition behavior of a solute in the ATPS, the analysis of solute partition behavior in practical applications was termed Solvent Interaction Analysis (SIA) [3].

The partition behavior of any solute between the two co-existing phases in an ATPS is quantified by its partition coefficient, which is defined as the ratio of the solute concentration in the top phase to its concentration in the bottom phase. The partition coefficient of a given protein can be used as a numerical index of the protein structure. Some of the typical analytical applications of ATPS partitioning for proteins in vitro include analyses of protein aggregation, protein–protein interactions, protein–ligand interactions, conformational changes, and chemical changes [1,4,5,6]. The partition behavior of a biological macromolecule in an ATPS of a fixed composition is determined by the nature and arrangement of the solvent-accessible groups. This arrangement is determined by the three-dimensional structure and conformation of the macromolecule. As a result, changes in the partition behavior of a protein in any given ATPS are generally a result of changes in the protein’s primary, secondary, tertiary, and/or quaternary structures, which can be due to truncation, single- or multiple-point mutations, posttranslational modifications (PTMs), and/or conformational changes. These changes can be induced by binding other molecules, aggregation, or the formation of breach of interactions with a partner protein in biological fluid [3].

Partitioning of solutes in an ATPS is driven by the hydrogen bonding, electrostatic, and dipole–dipole interactions of their solvent-exposed groups within the aqueous media [3,4,5,6,7,8,9,10,11,12,13,14,15,16]. Contributions from these different types of interactions have been evaluated for small organic compounds along with biological macromolecules [1,2,3]. The most fundamental finding thus far from studies of different types of solute–solvent interactions of simple organic compounds and proteins is that all water-soluble compounds are impacted by changes in their environment’s composition. This, in turn, may lead to modifications of the relative contributions of the different types of their interactions with the solvent into the solute partition coefficient.

Here, we present the analysis of the partition behaviors of single- and double-point mutants of bacteriophage T4 lysozyme [17] and staphylococcal nuclease A [18] in three ATPSs with the same polymer content but different salt additives/phosphate buffer quantities. We provide evidence that Solvent Interaction Analysis (SIA) signatures, determined from protein partitioning in ATPSs, can be used as a simple numerical descriptor of multiple structural and dynamic features of query proteins. We aim to use these relationships between protein partition coefficients and specific solvent properties of ATPSs as a method to identify mutants with specific structural characteristics [19].

## 2. Results

The partition coefficient, or K-value, represents the interactions between the solvent-exposed groups of a protein with two different solvent environments in a given ATPS [3]. This K-value can be used as a general-purpose numerical index to characterize the 3D structure of the partitioned protein; however, different structural changes in a protein can result in the same change in K in an ATPS [3]. We address this issue by combining multiple K-values for a single protein partitioned in a set of different ATPSs. The resulting partition coefficients can be combined to construct a vector, which is a numerical signature sensitive to single-point mutation, misfolding, aggregation, and interaction with binding partners [4,20]. The SIA signature represents a normalized Euclidian distance for various mutant proteins compared to a reference case (here, the wild type of the same protein under evaluation). These distances are calculated using logarithms of partition coefficients by utilizing the following equation:

(1) $$d_{i,0}=\sqrt{\sum_{j=1}^{n\sum }{\left(\frac{{logK}_{i,j}-{logK}_{0,j}}{{logK}_{0,j}}\right)}^{2}\frac{s_{j}}{s_{max}}}$$

where the distance d_i,0_ is calculated between any i-th mutant signature and the reference mutant signature for n aqueous systems (three for this case). The under-script j denotes the j-th ATPS, s_j_ is the normalized standard deviation in system j, and S_max_ is the largest standard deviation value across all aqueous two-phase systems.

The data reported in Table 1 and Table 2 represent a set of structural signatures corresponding to the staphylococcal nuclease A and T4 lysozyme mutants, respectively. The distance data reported in Table 1 can be interpreted individually for each mutant as a measure of the overall similarity between the conformation induced by a single-point mutation and a wild-type protein. 

**Figure A1:**
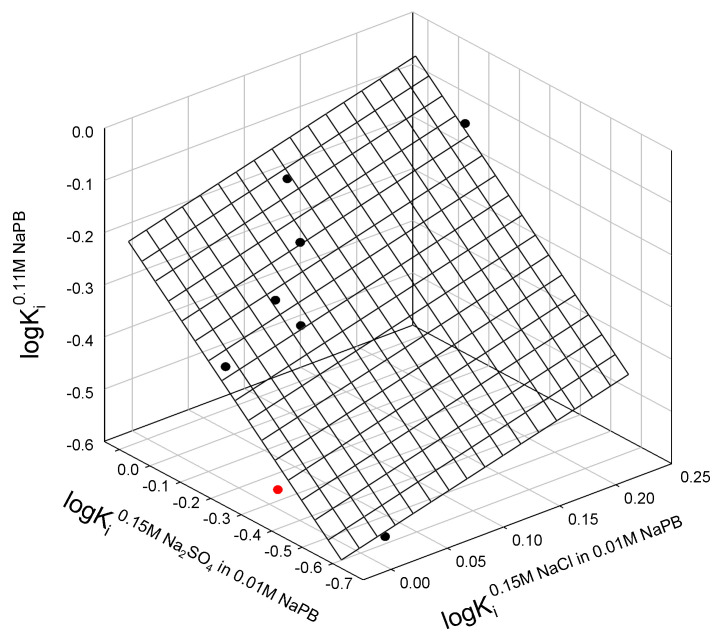
Relationship between partition coefficients of staphylococcal nuclease A mutants in three different ATPSs with ionic composition indicated. The single outlier is L108V mutant, shown in red.

**Figure A2:**
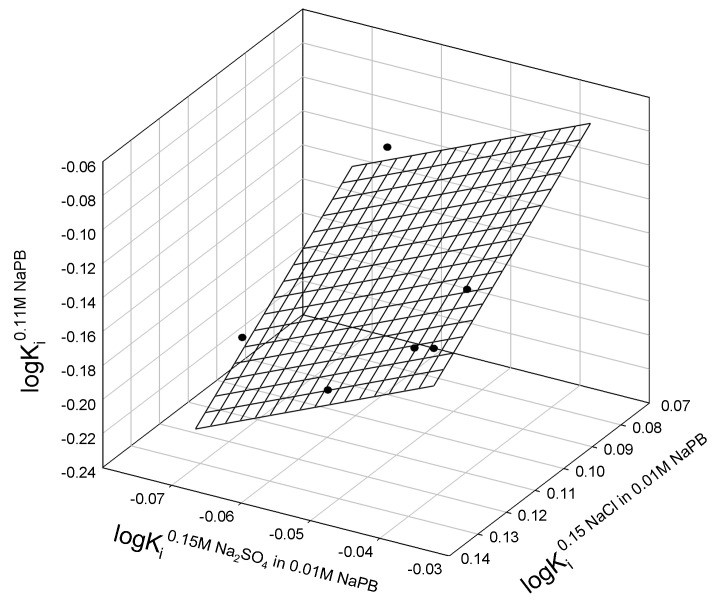
Relationship between the logarithms of partition coefficients of different mutants of lysozyme T4 in the ATPSs with different ionic compositions.

**Figure A3:**
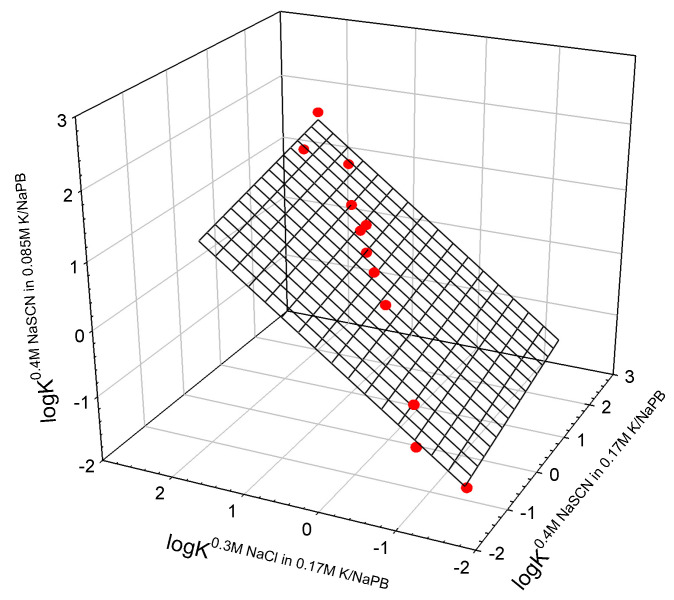
Relationship between logarithms of partition coefficients of different proteins (chymotrypsin, chymotrypsinogen, bovine hemoglobin, lactoglobulin A, lactoglobulin B, hen egg lysozyme, papain, ribonuclease A, ribonuclease B, subtilisin A, and trypsin) in the ATPSs with different ionic compositions (data from [28]).

Analysis of the data presented in Table 1 indicates a linear relationship (Equation (A1)) between the logarithms of the protein mutants’ partition coefficients in the ATPSs of different ionic compositions, illustrated by Figure A1 in the Appendix A. Similarly, the partition coefficients, SIA signatures, and thermodynamic data for lysozyme T4 mutants are listed in Table 2. Again, we observe a relationship between the partition coefficients of lysozyme T4 mutants in the same ATPSs, similar to that observed for staphylococcal nuclease A (Equation (A2)), illustrated by Figure A2 in the Appendix A. It is important to note that a correlation was previously reported between partition coefficients of 12 globular proteins in three different ATPSs with distinct ionic compositions, similar to those described here [22]. This relationship is also shown in the Appendix A in Figure A3 and Equation (A3).

**Table 2:** Partition coefficients, SIA signatures, and structural parameters of bacteriophage T4 lysozyme mutants adapted from [17,23]. ATPS #1 Dextran-Ficoll-0.15 M NaCl-0.01 M NaPB; ATPS #2 Dextran-Ficoll-0.15 M Na_2_SO_4_-0.01 M NaPB; ATPS #3 Dextran-Ficoll-0.11 M NaPB.

Mutant	ATPS #1	ATPS #2	ATPS #3	Signature	T_m_ ^a^	ΔΔG ^b^
C54T/C97A	1.30 ± 0.02	0.90 ± 0.02	0.68 ± 0.02	0.00	65.80	-
G113E	1.19 ± 0.02	0.86 ± 0.02	0.76 ± 0.01	1.33	65.99	0.3
T115A	1.32 ± 0.02	0.88 ± 0.02	0.64 ± 0.01	0.47	65.37	−0.14
T115A/S117A	1.30 ± 0.02	0.85 ± 0.01	0.65 ± 0.01	0.87	68.64	0.95
S117A/R119A	1.28 ± 0.02	0.91 ± 0.01	0.73 ± 0.01	1.13	69.17	1.16
R119A	1.35 ± 0.02	0.92 ± 0.02	0.73 ± 0.01	0.53	65.27	−0.18

^a^ T_m_—melting temperature (°C) of the bacteriophage T4 lysozyme mutants; ^b^ ΔΔG—difference between the free energy of unfolding of the mutant T4 lysozyme versus the wild-type protein.

**Figure 1:**
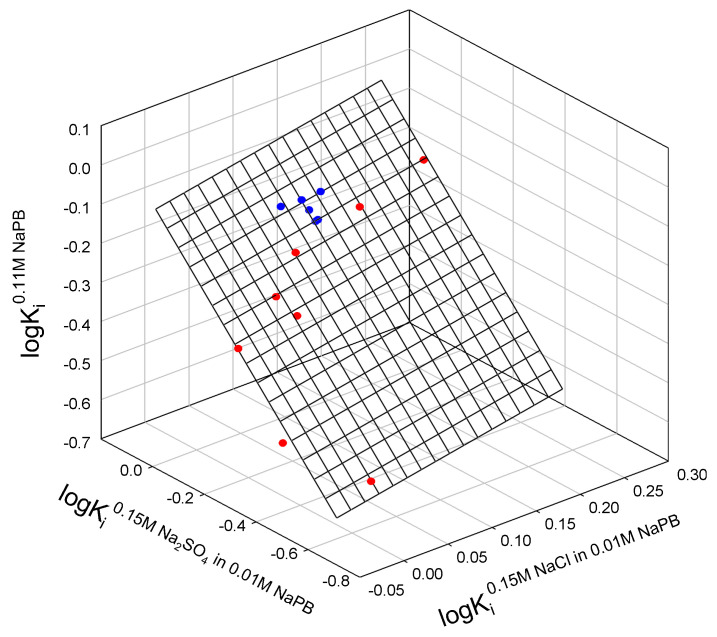
Relationship between the logarithms of partition coefficients of different mutants of staphylococcal nuclease (red), lysozyme T4 (blue) in the ATPSs with different ionic compositions.

Taken together, the partition coefficients for all mutants of both staphylococcal nuclease A and bacteriophage T4 lysozyme can be correlated, as shown in Figure 1. Interestingly, we observe a single outlier in this relationship, nuclease mutant L37I.

The relationship observed in Figure 1 is described as:

logK_i_^0.11M NaPB^ = −0.17_±0.03_ − 0.4_±0.20_ logK_i_^0.15M NaCl in 0.11M NaPB^ + 0.78_±0.09_ logK_i_^0.15M Na2SO4 in 0.11M NaPB^ (2)

N = 14; r^2^ = 0.9424; SD = 0.039; F = 89.9

where K_i_^0.11M NaPB^, K_i_^0.15M NaCl in 0.11M NaPB^, and K_i_^0.15M Na2SO4 in 0.11M NaPB^ are partition the coefficients of the i-th protein in ATPSs with ionic composition indicated; N is the number of mutants fitting the relationship; r^2^ is correlation coefficient; SD—standard deviation; F is the variance ratio. 

**Figure 2:**
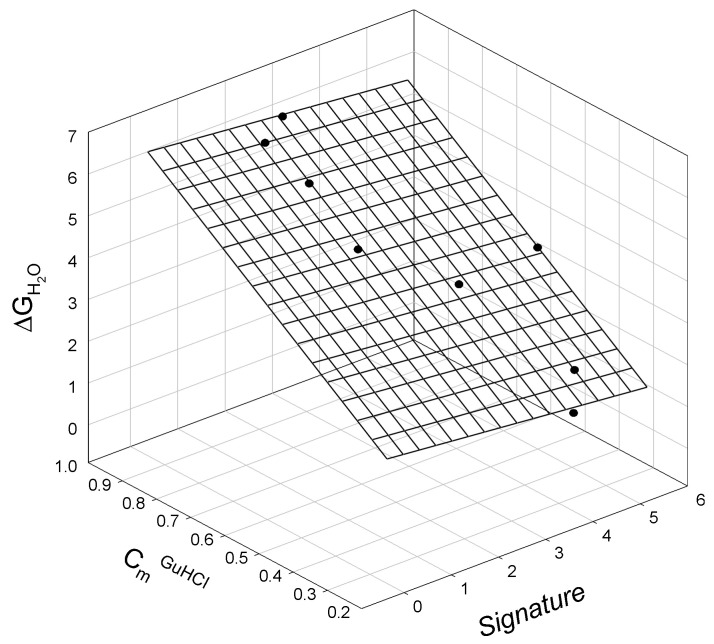
The linear relationship between the free energy of staphylococcal nuclease mutant unfolding, ΔG_H2O_, mutant signature, and the concentration of guanidine hydrochloride, C_m_^GuHCl^, at which half of the protein molecules are unfolded.

When comparing the thermodynamic data for staphylococcal nuclease A and bacteriophage T4 lysozyme mutants to their corresponding SIA signatures (calculated via Equation (A1) and Equation (A2), respectively), we again observe linear relationships. The relationship between the GuHCl-induced unfolding of staphylococcal nuclease mutants, ΔG_H2O_, in terms of the corresponding mutant signature (calculated using Equation (A1)) and the guanidine hydrochloride concentration, at which half of the protein molecules are unfolded, C_m_^GuHCl^, is graphically illustrated in Figure 2 and is described as:

ΔG_H2O_ = 0.56_±0.33_ − 0.12_±0.06_ Signature + 6.2_±0.30_ C_m_^GuHCl^ (3)

N = 8; r^2^ = 0.9953; SD = 0.14; F = 530

where all parameters are defined above.

**Figure 3:**
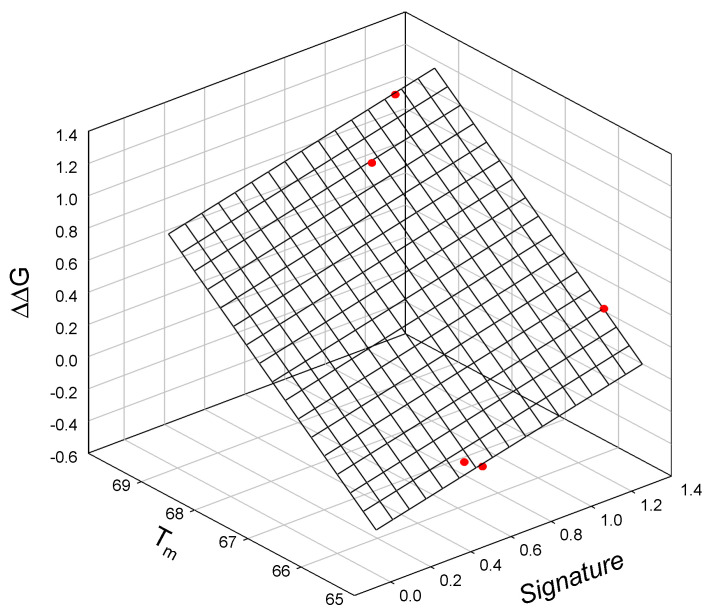
The linear relationship between the free energy of unfolding of the lysozyme T4 mutant relative to TA (C54T/C97A), mutant signature, and the melting temperature T_m_ of the mutants.

Analysis of the thermodynamic data for lysozyme T4 mutants shows that the free energy of unfolding is linearly related to the mutant SIA signature (calculated using Equation (A2)), the melting temperature T_m_ of the mutant protein, and the change in the free energy of the unfolding of the mutant protein relative to TA (C54T/C97A) [23]. This observed relationship is illustrated graphically in Figure 3 and is described as:

ΔΔG = −19.6_±0.40_ + 0.31_±0.03_ Signature + 0.295_±0.01_T_m_ (4)

N = 5; r^2^ = 0.9995; SD = 0.02; F = 1846

where all parameters are defined above.

Due to the observed relationships between previously published thermodynamic data and our calculated SIA signatures for mutants of both staphylococcal nuclease A and bacteriophage T4 lysozyme, we decided to probe the solvent accessibility of these mutants. Solvent-accessible surface area (SASA, also known as the Lee–Richards molecular surface) represents a geometric measurement of the surface area of a biomolecule that is accessible to the solvent molecules. This concept was introduced in 1971 by B.K. Lee and Frederic M. Richards (1925–2009) to distinguish buried versus accessible side chains and predict the macromolecule’s 3D conformation [24]. For any given protein, SASA is estimated computationally using the atomic coordinates of its available structure by rolling a “probe”, typically a sphere with a diameter of 1.4 Å (the size of a water molecule), over the protein surface. The probe rolls over the van der Waals surfaces of the exposed atoms as it cannot make contact with the deeply buried atoms. As a result of this analysis, the solvent-accessible surface is determined as the sum of all van der Waals radii plus the probe’s radius. Therefore, SASA represents a geometric measurement of a protein’s structure regarding its static accessibility to water molecules.

The SIA signature reflects the interactions between a solute (more precisely, the solute surface) with an aqueous solvent, and since a protein surface contains both polar and non-polar/apolar residues, we checked the presence of a correlation between the signature and calculated total, polar, and apolar SASA for the sets of the staphylococcal nuclease A and T4 lysozyme mutants.

Table 3 presents the calculated SASAs evaluated using predictions of 3D-structural models for the wild-type (WT) and mutant forms of staphylococcal nuclease A.

**Figure 4:**
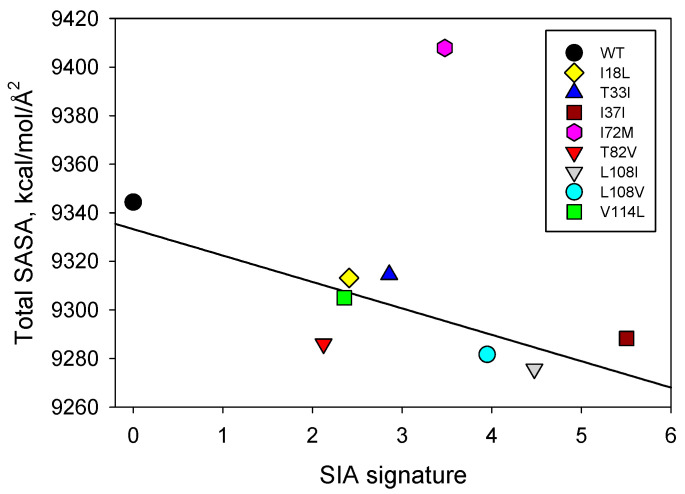
Correlation between the effects of point mutations on the total SASA of the staphylococcal nuclease A and SIA signatures of these mutants.

Figure 4 indicates that mutation-induced changes in the total SASA of the staphylococcal nuclease A are linearly correlated with the SIA signature, apart from mutant I72M. This relationship indicates that the partitioning of this protein and its interactions with the solvent are connected to its SASA. Interestingly, we see the largest difference in both apolar and total SASA between the WT staphylococcal nuclease A and mutant I72M, which is the single outlier. Although this appears to be a simple substitution of hydrophobic amino acids, methionine, unlike most other hydrophobic residues (e.g., isoleucine), is sulfur-containing and does not behave like a typical apolar amino acid. This specific mutation may lead to very specific perturbations in how nuclease interacts with the aqueous solvent in comparison to the other mutants examined here [25].

**Figure A4:**
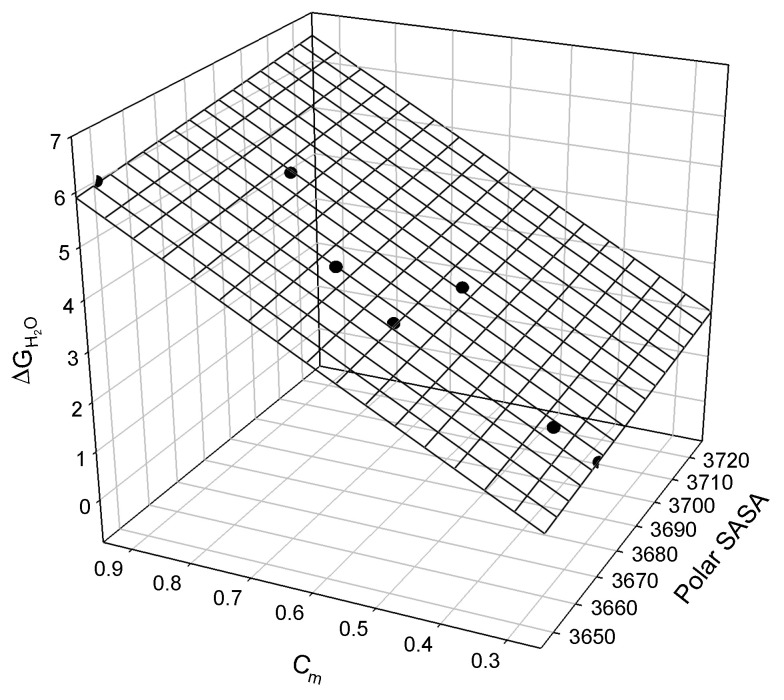
The linear relationship between the free energy of staphylococcal nuclease mutants’ denaturation, ΔG_H2O_, polar surface-accessible surface area, polar SASA, and the concentration of guanidine hydrochloride, c_m_^GuHCl^, at which half of the protein molecules are unfolded (see in [27]).

The polar SASA predicted values qualitatively correlate with the free energy of staphylococcal nuclease mutants’ denaturation, ΔG_H2O_, and the concentration of guanidine hydrochloride, C_m_^GuHCl^. This linear relationship is shown in Figure A4 and Equation (A4).

**Figure 5:**
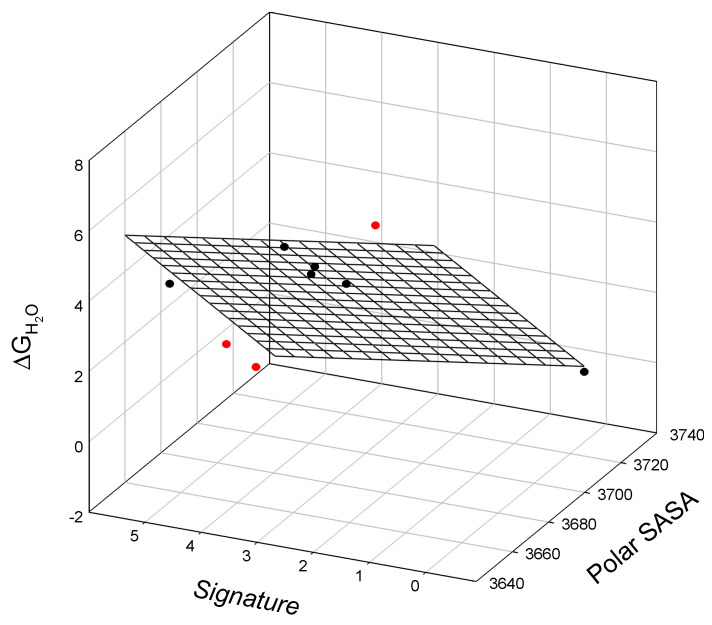
The linear relationship between the free energy of denaturation of staphylococcal nuclease mutants, ΔG_H2O_, their polar surface-accessible surface area, polar SASA, and signature. Outliers below (L108V and L108I) or above the surface (I18L) are shown in red.

When we compare the thermodynamic data for staphylococcal nuclease A mutants to both the SIA signature calculations and predicted SASA values, we again observe a linear relationship. The correlation between the polar SASA, SIA signature, and the free energy of staphylococcal nuclease mutant denaturation, ΔG_H2O_, is graphically illustrated in Figure 5.

The observed relationship is graphically illustrated in Figure 5 and is described as:

ΔG_H2O_ = 312_±48_ − 0.08_±0.01_ Polar SASA − 0.23_±0.18_ Signature (5)

N = 6; r^2^ = 0.9650; SD = 0.53; F = 41.4

where all parameters are defined above. Here, we observe three outliers in this linear relationship, L108V, L108I, and I18L.

**Figure 6:**
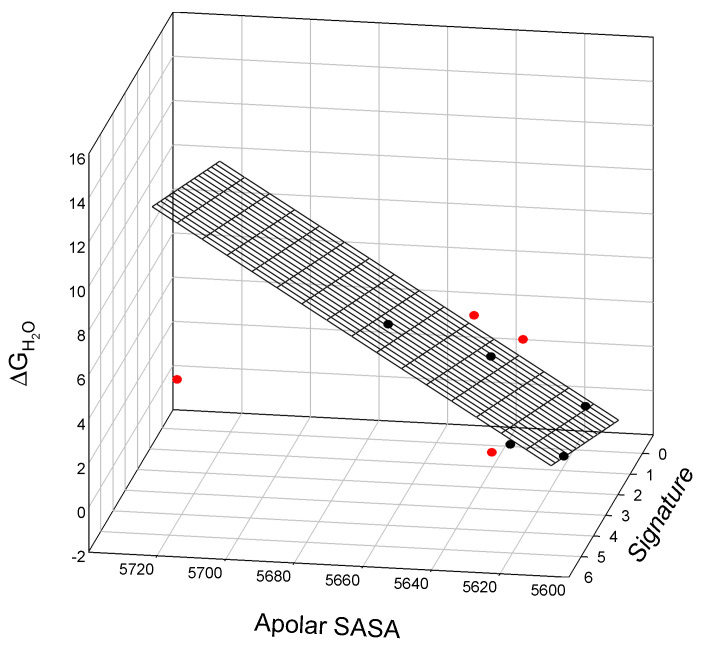
The linear relationship between the free energy of denaturation, ΔG_H2O_, apolar surface-accessible surface area, Nonpolar SASA, and Signature of staphylococcal nuclease mutants. Outliers located below (I72M and L108V) or above the surface (I18L and T82V) are shown in red.

We also observe a linear relationship between the nonpolar SASA values, SIA signature, and free energy of denaturation, ΔG_H2O_, of staphylococcal nuclease mutants. This relationship is illustrated in Figure 6 and is described as:

ΔG_H2O_ = −520_±91_ + 0.09_±0.02_ Nonpolar SASA + 0.6_±0.20_ Signature (6)

N = 5; r^2^ = 0.9679; SD = 0.55; F = 30.2

where all parameters are defined above. The outliers for this relationship are I18L, I72M, T82V, and L108V.

Table 4 presents the calculated solvent-accessible surface area (SASA) evaluated using the AlphaFold predictions of 3D-structural models for the wild-type (WT) and mutant forms of bacteriophage T4 lysozyme.

**Figure 7:**
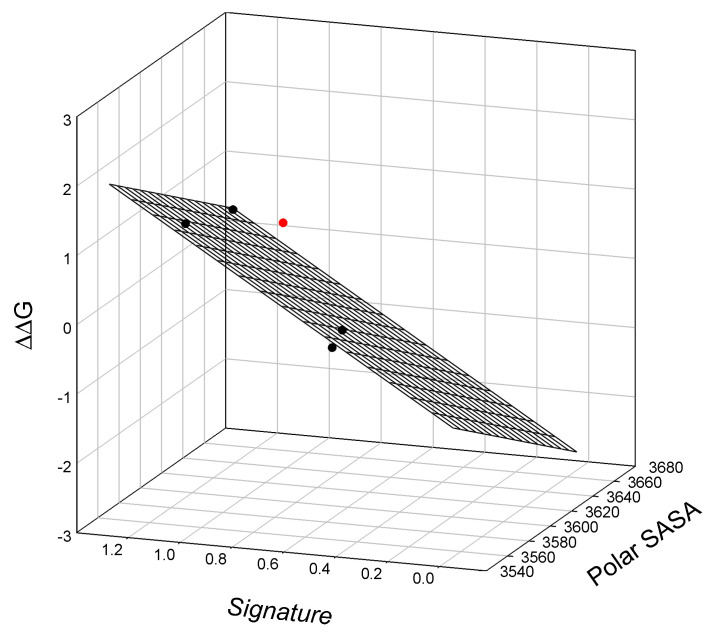
The linear relationship between the free energy of the unfolding of lysozyme T4 mutants relative to TA (C54T/C97A), polar solvent-accessible surface area, and signatures of the mutants. Outlier T115A/S117A (located above the surface) is shown in red.

**Figure A5:**
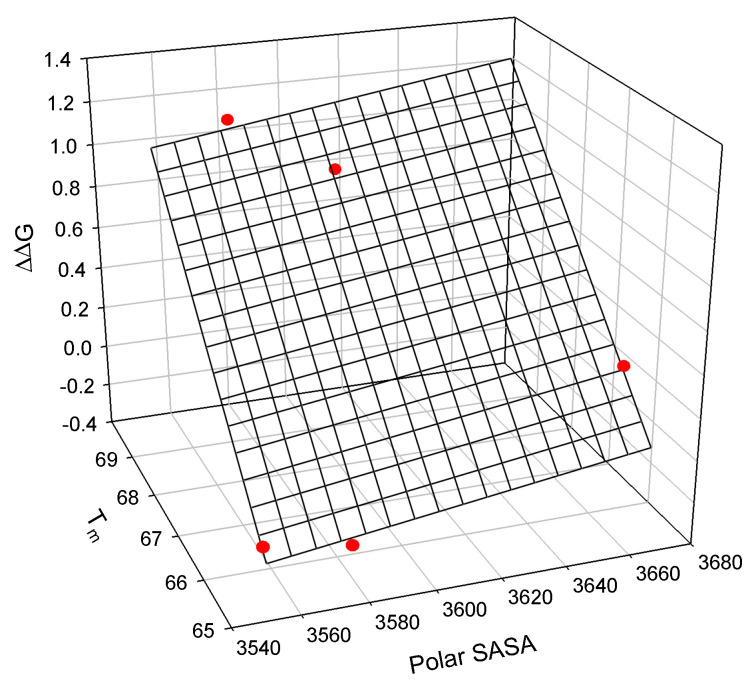
The linear relationship between the free energy of unfolding of the T4 lysozyme mutants relative to TA (C54T/C97A), the mutants’ polar solvent-accessible surface area, and the mutants’ melting temperature Tm [29].

Again, our analysis shows a linear relationship between the SIA signature, polar SASA, and the thermodynamics of lysozyme T4 mutants, graphically illustrated in Figure A5 and calculated via Equation (A5). Similarly, we show a linear relationship between the SIA signature, polar SASA, and the free energy of lysozyme T4 mutant denaturation, ΔG_H2O_, as illustrated graphically in Figure 7.

The observed relationship graphically illustrated in Figure 7 is described as:

ΔΔG = 47.8_±7.4_ − 0.014_±0.00_ Polar SASA + 2.3_±0.20_ Signature (7)

N = 4; r^2^ = 0.9886; SD = 0.115; F = 43.2

where all parameters are defined above. Here, we find a single outlier in this relationship, mutant T115A/S117A.

## 3. Discussion

Data collected in this study provide vital support to the hypothesis that the SIA signatures determined from protein partitioning in ATPSs can be used as simple numerical descriptors related to the structural and dynamic features of query proteins. Here, we show that for sets of mutants of staphylococcal nuclease A and bacteriophage T4 lysozyme, the calculated SIA signatures correlate well with the free energy of protein unfolding or denaturation, along with the calculated solvent-accessible surface area (SASA) values derived from structural models (see Figure 5, Figure 6 and Figure 7). Although SASAs and SIA signatures both characterize interactions of a protein with a solvent, these two parameters are principally different. SASA is calculated based on the unique 3D structure; therefore representing a static geometrical parameter, whereas the SIA signature reflects the dynamic behavior of a protein in solution (i.e., various ATPSs).

**Figure 8:**
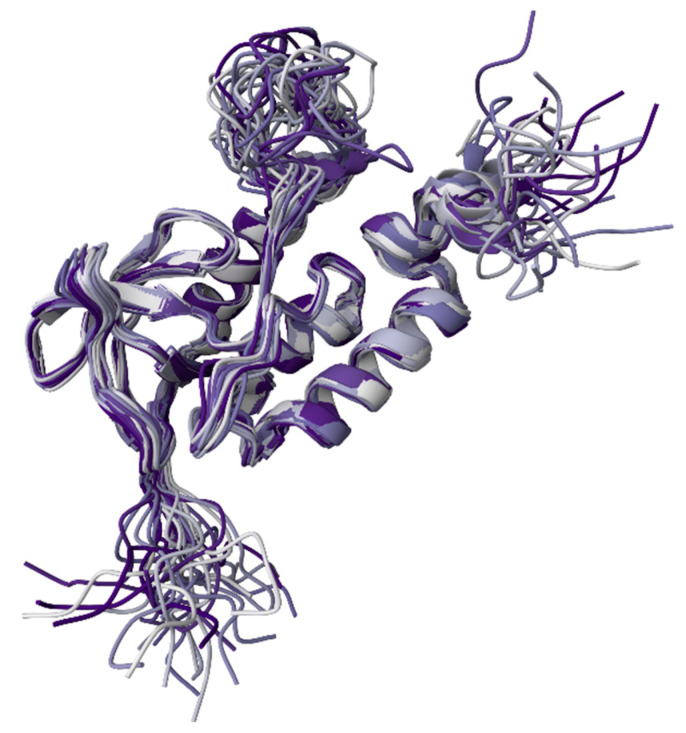
Solution NMR structure of the staphylococcal nuclease A H124L (PDB ID: 1JOR; [44]).

Within aqueous media, protein SASA is correlated with protein hydrophobicity [26,27,28] and the number of native intraprotein contacts [29,30]. Specifically, for globular proteins, a very tight correspondence was found between the SASA of folded, crystallizable, monomeric proteins and their molecular weights [31,32,33,34]. Furthermore, the intrinsic flexibility of a protein has been shown to be controlled by the total amount of surface area buried in its folded structure [32]. A systematic analysis of calculated SASA values along with intraprotein and protein–water hydrogen bonds conducted for 55 single-chain globular proteins from four different structural classes, 16 multichain proteins, and 4 macromolecular complexes revealed that SASAs and hydrogen bonds are linearly correlated [35]. In fact, irrespective of the protein structural class, the number of protein–water hydrogen bonds per unit SASA was shown to range from 3 to 4, whereas the number of intramolecular hydrogen bonds per unit SASA varied between 0.75 to 2 [35]. Transient hydrogen bonding between any protein of interest and aqueous solvent is important to note due to its importance for various biological processes, such as protein folding, stabilization of protein structure, and biomolecular recognition [35,36,37,38,39,40,41,42,43]. To further illustrate this, Figure 8 represents the solution NMR structure of a staphylococcal nuclease A ensemble with a rather dynamic structure characterizes this protein consisting of several flexible regions. From this visual representation, it is clear that the members of this conformational ensemble would be characterized by different SASAs. In fact, although the overall root-mean-square deviation from the mean atomic coordinates for the backbone heavy atoms of nuclease is 0.46 ± 0.05 Å, the N- and C-terminal regions (residues 1–9 and 142–149) as well as a loop (residues 40–53) of this protein are highly flexible.

Despite these limitations, SASA can still reflect some degree of protein folding and conformational stability [45]. Therefore, mutation-induced changes in SASA are expected to correlate with the changes in protein conformational stability for a given protein. This is based on the following: smaller SASA corresponds to a more compact protein, which is expected to be more conformationally stable. Our analyses revealed that SASA and SIA signatures are correlated, as illustrated in Figure 5, Figure 6 and Figure 7. This correlation is impactful as SASA represents a geometric measure of a protein’s structure regarding its static accessibility to water molecules, whereas the SIA signature reflects the interactions between a protein’s surface with an aqueous solvent. Use of the SIA signature as a physicochemical method to detect subtle structural modifications (here, single- and double-point amino acid substitutions) is further strengthened by the strong correlation of SIA signatures with conformational stabilities within the sets of the staphylococcal nuclease and T4 lysozyme mutants. We show a strong correlation between SIA signatures and the free energy of staphylococcal nuclease mutants unfolding, ΔG_H2O_, polar SASA, and the concentration of guanidine hydrochloride, C_m_^GuHCl^, at which half of the protein molecules are unfolded (Figure 6, Equation (6)), and the free energy of unfolding of the lysozyme T4 mutant relative to TA, polar SASA of mutants, and the melting temperature T_m_ of the mutants (Figure A5, Equation (A5)).

## 4. Materials and Methods

### 4.1. Polymers and Salts

Ficoll-70 (Lot 128K1136), with an average molecular weight of 70,000 Da, was purchased from Sigma-Aldrich (St. Louis, MO, USA). Dextran-75 (Lot 119945), with an average molecular weight (Mw) of 75,000 Da by light scattering, was obtained from USB Corporation (Cleveland, OH, USA). All inorganic salts were obtained from Sigma-Aldrich.

### 4.2. Proteins

Mutants of staphylococcal nuclease were kindly provided by Professor Wesley E. Stites (University of Arkansas) [21]. Mutants of the phage T4 lysozyme were provided by Professor Brian W. Matthews (University of Oregon) [23].

### 4.3. Aqueous Two-Phase Systems

Aqueous two-phase systems (ATPSs) were prepared by dispensing appropriate amounts of aqueous stock solutions (prepared as described in [1,2]) of polymers, stock buffer solutions, salt additive(s), and water into 1.2 mL Simport microtube strips using a Hamilton Company (Reno, NV, USA) ML-4000 four-probe liquid-handling workstation. Appropriate amounts of each component were added to achieve the ionic and polymer composition required for 0.5 g final systems after the sample addition. A sodium phosphate buffer (NaPB) solution at pH 7.4 was prepared by mixing 21.7 g of Na_2_HPO_4_·7H_2_O with 2.6 g of NaH_2_PO_4_·H_2_O in up to 200 mL of water.

### 4.4. Partitioning

Partitioning experiments were performed at room temperature (approximately 23 °C) using the custom-built Automated Signature Workstation, ASW (Analiza, Inc., Cleveland, OH, USA). This system is custom-built to integrate an ML-4000 liquid handler (Hamilton Company, Reno, NV, USA) with an FL600 fluorescence microplate reader (Bio-Tek Instruments, Winooski, VT, USA), and a UV-VIS microplate spectrophotometer (SpectraMax Plus 384, Molecular Devices, Sunnyvale, CA, USA). All protein solutions were prepared in water at 1–5 mg/mL concentrations. Varied amounts (e.g., 0, 15, 30, 45, 60, and 75 μL) of protein solution and the corresponding amounts (e.g., 75, 60, 45, 30, 15, and 0 μL) of water were added to a set of the same ATPSs containing a free volume equivalent to the sum of protein solution and water volumes (e.g., 100 μL). After sample and water addition, the systems were vortexed in a Multipulse vortexer and centrifuged (Jouan, BR4i, Thermo Fisher Scientific, Waltham, MA, USA) for 60 min at 3500× *g* at 23 °C to accelerate phase settling. The top and bottom phases in each system were removed and aliquoted and the interface was discarded. Aliquots from the top and bottom phases were dispensed in duplicate for analysis.

For the analysis of protein partitioning, aliquots of 30 μL from both phases were transferred and diluted with water up to 70 μL into 96-well microplate wells. The microplate was then sealed, shortly centrifuged (2 min at 1500 rpm), followed by moderate shaking for 45 min in an incubator at 37 °C. After incubation, 250 μL of o-phthaldialdehyde reagent was added to each microwell followed by moderate shaking for 4 min at room temperature. Fluorescence was determined using a 360 nm excitation filter and a 460 nm emission filter, with a sensitivity setting of 100–125.

The partition coefficient, or K-value, for each protein was determined as the slope of the concentration (i.e., fluorescence intensity) in the top phase plotted as a function of the concentration in the bottom phase. Two to four independent partitioning experiments were conducted, and K-values were averaged. The deviation from the average K-values was always below 3% and, in most cases, lower than 1%.

### 4.5. Calculation of Solvent-Accessible Surface Areas

Structural models of the T4 lysozyme (UniProt #P00720) and the staphylococcal nuclease A (UniProt #P00644, residues 83–231) and their related mutants were generated by ColabFold v1.5.5 [46] and then uploaded individually to GETAREA (https://curie.utmb.edu/getarea.html; accessed 15 July 2024) [47] for calculation of Solvent-Accessible Surface Areas (SASAs). In this study, we evaluated total, polar, and apolar SASAs for the WT staphylococcal nuclease A and its I18L, T33I, L37I, I72M, T82V, L108V, L108I, and V114L mutants, and for the WT T4 lysozyme and its G113E, T115A, T115A/S117A, S117A/R119A, and R119A mutants, which were subjected to SIA analysis.

## Figures and Tables

**Table 1:** Partition coefficients, SIA signatures, and thermodynamic parameters derived from guanidine hydrochloride (GuHCl)-induced unfolding experiments for mutants of staphylococcal nuclease A [21]. ATPS #1 Dextran-Ficoll-0.15 M NaCl-0.01 M NaPB; ATPS #2 Dextran-Ficoll-0.15 M Na_2_SO_4_-0.01 M NaPB; ATPS #3 Dextran-Ficoll-0.11 M NaPB.

Mutant	ATPS #1	ATPS #2	ATPS #3	Signature	*m*_GuHCl_ ^a^	*C*_m_ ^b^	Δ*G*_H2O_ ^c^
WT	0.97 ± 0.01	0.42 ± 0.02	0.29 ± 0.02	0.00	6.53	0.8	5.4
I18L	1.14 ± 0.03	0.64 ± 0.04	0.46 ± 0.05	2.41	6.45	0.8	5.2
T33I	1.10 ± 0.02	0.68 ± 0.03	0.52 ± 0.01	2.86	6.34	1.0	6.0
L37I	1.33 ± 0.10	1.25 ± 0.12	0.63 ± 0.06	5.50	6.00	0.6	3.4
I72M	1.18 ± 0.07	0.73 ± 0.07	0.62 ± 0.02	3.48	6.99	0.5	3.6
T82V	0.96 ± 0.03	0.60 ± 0.03	0.45 ± 0.04	2.12	6.51	0.9	5.9
L108I	1.73 ± 0.24	0.86 ± 0.05	0.73 ± 0.14	4.48	6.02	0.3	2.0
L108V	1.43 ± 0.06	0.79 ± 0.01	0.67 ± 0.02	3.95	6.31	0.3	1.5
V114L	1.05 ± 0.06	0.25 ± 0.07	0.26 ± 0.03	2.36	6.48	0.7	4.3

^a^ *m*_GuHCl_—change in free energy for the change in guanidine hydrochloride concentration (in kcal·mol^−1^·M^−1^) with error estimates of 0.09 kcal·mol^−1^·M^−1^; ^b^ *C*_m_—concentration of guanidine hydrochloride (in molar units) at which half of the protein is denatured with error estimates of 0.01 M; ^c^ Δ*G*_H2O_—free energy difference (in kcal/mol) between native and denatured states without denaturant. Errors are estimated to be 0.1 kcal/mol.

**Table 3:** Solvent-Accessible Surface Area (SASA) values for wild type and mutants of staphylococcal nuclease A.

Mutant	Apolar SASA, kcal/mol/Å^2^	Polar SASA, kcal/mol/Å^2^	Total SASA, kcal/mol/Å^2^
WT	5616.60	3727.75	9344.35
I18L	5626.19	3687.07	9313.21
T33I	5663.88	3650.57	9314.50
L37I	5618.81	3669.40	9288.21
I72M	5723.19	3684.65	9407.84
T82V	5641.46	3644.60	9286.05
L108V	5629.76	3651.87	9281.63
L108I	5606.91	3668.80	9275.71
V114L	5635.73	3669.26	9304.99

**Table 4:** Solvent-Accessible Surface Area (SASA) values for the wild-type and mutants of T4 lysozyme.

Mutant	Apolar SASA, kcal/mol/Å^2^	Polar SASA, kcal/mol/Å^2^	Total SASA, kcal/mol/Å^2^
WT	5066.36	3595.81	8662.36
G113E	5047.41	3668.85	8716.27
T115A	5066.34	3552.63	8619.03
T115A/S117A	5048.58	3605.60	8654.18
S117A/R119A	5049.08	3576.61	8625.69
R119A	5053.94	3577.71	8631.65

## Data Availability

The raw data supporting the conclusions of this article will be made available by the authors upon request.

## Appendix A

The relationship in Figure A1 is described as:

logK_i_^0.11M NaPB^ = −0.25_±0.05_ + 0.6_±0.4_ logK_i_^0.15M NaCl in 0.11M NaPB^ + 0.6_±0.1_ logK_i_^0.15M Na2SO4 in 0.11M NaPB^ (A1)

N = 8; r^2^ = 0.8904; SD = 0.063; F = 20.3

where K_i_^0.11M NaPB^, K_i_^0.15M NaCl in 0.11M NaPB^, and K_i_^0.15M Na2SO4 in 0.11M NaPB^ are partition coefficients of i-th protein in ATPSs with ionic composition indicated; N is the number of mutants fitting the relationship; r^2^ is correlation coefficient; SD—standard deviation; F is the variance ratio.

The relationship in Figure A2 is described as:

logK_i_^0.11M NaPB^ = 0.10_±0.09_ − 1.5_±0.6_ logK_i_^0.15M NaCl in 0.11M NaPB^ + 1.8_±0.7_ logK_i_^0.15M Na2SO4 in 0.11M NaPB^ (A2)

N = 6; r^2^ = 0.7491; SD = 0.02; F = 4.5

where K_i_^0.11M NaPB^, K_i_^0.15M NaCl in 0.11M NaPB^, and K_i_^0.15M Na2SO4 in 0.11M NaPB^ are partition coefficients of i-th protein in ATPSs with ionic composition indicated; N is the number of mutants fitting the relationship; r^2^ is correlation coefficient; SD—standard deviation; F is the variance ratio.

The linear relationship shown in Figure A3 is described as:

logK_i_^0.4M NaSCN−0.085M K/NaPB^ = −0.16_±0.04_ + 0.2_±0.1_ logK_i_^0.4M NaSCN−0.17M K/NaPB^ + 0.8_±0.1_ logK_i_^0.3M NaCl−0.17M K/NaPB^ (A3)

N = 12; r^2^ = 0.9918; SD = 0.11; F = 542

where K_i_^0.4M NaSCN−0.085M K/NaPB^, K_i_^0.4M NaSCN−0.17M K/NaPB^, and K_i_^0.3M NaCl−0.17M K/NaPB^ are partition coefficients of i-th protein in ATPSs with ionic composition indicated; N is the number of mutants fitting the relationship; r^2^ is correlation coefficient; SD—standard deviation; F is the variance ratio.

The observed relationship is graphically illustrated in Figure A4 and may be described as:

ΔG_H2O_ = −28_±21_ + 0.008_±0.006_ Polar SASA + 6.5_±0.28_ c_m_^GuHCl^ (A4)

N = 7; r^2^ = 0.9930; SD = 0.17; F = 283

where all parameters are defined above.

The observed relationship graphically illustrated in Figure A5 is described as:

ΔΔG = −28.7_±2.6_ + 0.0020_±0.0007_ Polar SASA + 0.30_±0.02_ T_m_ (A5)

N = 5; r^2^ = 0.9952; SD = 0.06; F = 208

where all parameters are defined above.
